# Glycosylation Targeting: A Paradigm Shift in Cancer Immunotherapy

**DOI:** 10.7150/ijbs.93806

**Published:** 2024-04-22

**Authors:** Xueting Ren, Shuai Lin, Feng Guan, Huafeng Kang

**Affiliations:** 1 Department of Oncology, the Second Affiliated Hospital of Xi'an Jiaotong University, Xi'an, Shaanxi, China.; 2 Key Laboratory of Resource Biology and Biotechnology in Western China, Ministry of Education, College of Life Sciences, Northwest University, Xi'an, Shaanxi, China.

## Abstract

Immunotherapy has shown great potential in cancer treatment. However, even with the intervention of techniques such as immune checkpoint inhibitor therapy, tumors can still achieve immune escape, leading to a low response rate. Abnormal glycosylation is a widely recognized hallmark of cancer. The development of a complex “glyco-code” on the surface of tumor cells can potentially influence the immune system's ability to monitor tumors and can impact the anti-tumor immune response. Therefore, abnormal glycosylation has emerged as a promising target for immunotherapy. Many recent studies have shown that targeted glycosylation can reshape the tumor microenvironment (TME) and promote the immune response, thereby improving the response to immunotherapy. This review summarizes how glycosylation affects anti-tumor immune function in the TME and synthesizes the latest research progress on targeted glycosylation in immunotherapy. It is hoped that by elucidating the basic laws and biological connotations of glycosylation, this review will enable researcher to thoroughly analyze the mechanism of its influence on the immune metabolic regulation network, which will provide a theoretical tool for promoting the clinical application of glycosylation codes.

## Introduction

In recent years, with continuous innovation in tumor treatment, fruitful results have been achieved, from traditional treatment methods (surgery, chemotherapy, and radiotherapy) to targeted drugs, and we have now entered a new era of immunotherapy. Unlike traditional treatments, immunotherapy utilizes cytokines, chemokines, and immune cells to reshape the tumor microenvironment (TME), thereby improving the anti-tumor efficacy and preventing recurrence 1. The development of monoclonal antibodies (mAbs), immune checkpoint inhibitors (ICIs), adoptive cell transfer therapy (ACT), and tumor vaccines has shown surprising therapeutic prospects for some cancers. In particular, the success of ICIs, represented by PD-1/PD-L1 mAbs, and of ACT, represented by chimeric antigen receptor T (CAR-T) cell therapy, in the field of anti-tumor therapy represents a new stage in tumor immunotherapy. However, owing to the influence of primary or acquired drug resistance caused by tumor immune escape, the vast majority of patients do not benefit from immunotherapy 2. Therefore, conducting an in-depth exploration of the immune escape mechanism and developing new strategies to overcome immune escape are the only ways to enhance the response efficiency of immunotherapy and make it more widely used.

Glycosylation is the most common and complex post-translational modification (PTM) that requires the coordination of different glycosyltransferases, glycosidases, nucleotide sugar transporters, and appropriate substrates 3. In recent years, changes in the surface glycosylation patterns of cancer cells have received extensive attention. Abnormal glycosylation is a marker for the occurrence and development of malignant tumors and plays an important role in regulating key carcinogenic processes (including malignant transformation, invasion, metastasis, angiogenesis, and immune escape) 4, 5. Notably, the regulation of tumor immunity by abnormal glycosylation not only leads to immunosuppression by changing the recognition of tumor cells by the immune system but also induces immune escape by affecting the binding of cell-surface glycosylation receptors to ligands 6. Tumor-associated glycosylation has been widely studied as a biomarker. The most common abnormal glycosylation modifications are O-glycan truncation, increased N-glycan branching, and changes in sialylation and fucosylation 4. For example, the interaction between sialoglycan and Siglec receptors contributes to the formation of an immunosuppressive TME by inducing the tumor-promoting phenotype of tumor-associated macrophages (TAMs), inhibiting the activation of natural killer (NK) cells and neutrophils, reducing dendritic cell (DC) maturation and antigen presentation, and inhibiting T cell responses 7. In addition, most immune checkpoint molecules are glycoproteins whose structure and biological functions are largely affected by glycosylation. For instance, N-glycosylation can reduce the proteasomal degradation of PD-L1 and maintain its interaction with PD-1, thereby inhibiting T cell immune escape 8, whereas O-GlcNAcylation can facilitate tumor immune evasion by suppressing the lysosomal degradation of PD-L1 9.

Many recent studies have shown that targeted glycosylation can improve tumor immunotherapy, offering a new clinical perspective for advancing cancer treatment 10. Hence, this study aims to review and summarize the research progress on the role of glycosylation in regulating tumor immunity and affecting immunotherapy and to provide objective reasoning and direction for further research on the prospect of targeting glycosylation to improve the efficacy of immunotherapy.

## O-glycosylation and immunotherapy

O-glycosylation is a PTM in which glycans are covalently bound to the hydroxyl groups of serine (Ser), threonine (Thr), or hydroxylysine (Hyl) in the polypeptide chain to form O-glycosidic bonds 11. O-glycosylation structures are complex and diverse, including O-GlcNAc modification; O-xylose-linked glycosaminoglycans; O-mannosylation of dystroglycan, cadherins, and protocadherins; O-fucosylation and O-glucosylation of Notch receptors; O-galactosylation of collagen; and, finally, the O-glycosylation modification of O-linked N-acetylgalactosamine (O-GalNAc), which is most abundant on membrane and secretory proteins 12.

GalNAc modification is initiated by up to 20 different polypeptide GalNAc-transferase isozymes (ppGalNAc-Ts) with different but partially overlapping substrate specificities 13. The initiation and extension of O-GalNAc glycosylation produces a single GalNAc (Tn antigen), which is further extended into different core structures as the backbone of complex O-GalNAc glycans 14. O-GalNAc modification affects protein folding, stability, transport, and protein interactions and participates in inflammatory responses, pathogenic microbial immune escape, cell adhesion, migration, apoptosis, and other key physiological and pathological processes 11. In tumor cells, changes in glycosylation sites and/or glycan structures often lead to the exposure of protein epitopes. In addition to the exposed polypeptide sequences, abnormal glycosylation can result in the production of novel glycans that subsequently become tumor-associated carbohydrate antigens 15. Elevated expression of truncated O-glycans, such as tumor-associated Tn and sTn, is often observed in malignant tumors such as breast cancer (BC) and colorectal cancer (CRC), which bind with c-type macrophage galactose-type lectin to evade immune surveillance, resulting in poor prognosis 16. Related studies have also shown that the binding of tumor-associated sTn antigens to lectins expressed on immune cells inhibits DC maturation and affects antigen presentation, thereby hindering T cell stimulation and activation and allowing for immune escape 17. In preclinical trials, a vaccine targeting sTn (Theratope) induced an effective antibody response and delayed BC growth in mice. However, in a large phase 3 clinical trial (NCT00003638) of patients with metastatic BC, Theratope did not affect the time to progression or overall survival (OS); nevertheless, the addition of Theratope to an endocrine therapy regimen may improve the clinical outcome of patients 18. MUC1 is a transmembrane glycoprotein with extensive O-GalNAc modifications. In tumors, the long O-GalNAc branch of MUC1 is truncated to form a classic tumor antigen, making it an emerging target for immunotherapy 19. MUC1 is overexpressed in various solid tumors, particularly BC, and has become the most relevant and important antigen in BC-targeted therapy. Studies have shown that the targeted O-GalNAc modification of MUC1 plays a role in changing the chemotherapy tolerance of BC and improving the efficacy of anti-tumor therapy 20. Mucin antigen vaccines are being actively used to treat various solid tumors. An MUC1 glycoprotein-targeted DC vaccine (CVac) has been shown to be safe and well tolerated in patients with ovarian cancer (OC). Compared with the standard of care, patients in second clinical remission (CR2) showed improved progression-free survival (18 vs. 13 months) and prolonged OS (>42 vs. 26 months), suggesting that targeting MUC1 is worthy of further study (NCT01068509) 21. In a phase 3 clinical trial of patients with non-small cell lung cancer (NSCLC), tecemotide, another vaccine against MUC1, combined with chemoradiotherapy showed no significant difference in OS compared to a placebo (NCT00409188) 22. Other vaccines targeting MUC1, such as ETBX-061, TG4010, and ImMucin, as well as other vaccines targeting glycosylation, are also in different clinical trial stages (Table 1). The Tn glycoform of MUC1 (Tn-MUC1) is a promising target for CAR therapy, and several CAR therapies targeting Tn-MUC1 are currently in preclinical research. Anti-Tn-MUC1 CAR-T cells reportedly showed specific targeted cytotoxicity and anti-tumor efficacy in T cell leukemia and pancreatic cancer xenograft models 23. In addition to directly targeting the O-glycosylation modification of glycoproteins, the regulation of some glycosyltransferases can lead to changes in the glycan pattern of target proteins, and changes in the function of related proteins are beneficial to tumor cell progression. Glycosyltransferase targeting is expected to become a new means for tumor treatment. Core-1-β1,3-galactosyltransferase (C1GALT1) is the key enzyme controlling the initiation of O-GalNAc glycosylation; it not only directly promotes cell survival and invasion but also contributes to tumor-mediated immune escape. When the expression of C1GALT1 is inhibited in head and neck cancer, it leads to a reduction in immunosuppressive macrophages and an enhanced killing effect of effector T cells, thereby reshaping the TME 24.

GlcNAcylation is an enzymatic process directed by N-acetyl-D-glucosamine (GlcNAc) glycosyltransferase (OGT), which transfers GlcNAc to proteins (Ser and Thr residues) in the cytoplasm and nucleus, and its removal is regulated by O-GlcNAcase (OGA) 25, 26. O-GlcNAcylation controls the development, activation, and differentiation of various T cell subsets at different stages through the dynamic coordination of OGT and OGA, which is essential for regulating the activation of homeostatic and mature B cells, as well as efficient germinal centers and antibody responses; regulating macrophage inflammation and antiviral responses; promoting the function of activated neutrophils; and inhibiting the activity of NK cells 27.

Recent studies have shown that PTM of the PD-L1 protein can regulate its stability and interactions with PD-1, thereby affecting anti-tumor immunotherapy of various solid tumors. Further elucidation of the molecular mechanisms that regulate PD-L1 expression is expected to provide new intervention targets for tumor treatment. OGT in exosomes derived from esophageal carcinoma stem cells (ECSCs) can be taken up by adjacent CD8^+^ T cells and increase the expression of PD-1 in CD8^+^ T cells, thereby inhibiting the killing of ECSCs by CD8^+^ T cells. When OSMI-1 was used to inhibit OGT activity, the self-renewal and immune escape abilities of ECSCs were inhibited 28. The lysosomal degradation of PD-L1 is mediated by intracellular endosomal sorting complexes required for transport (ESCRT). Hepatocyte growth factor-regulated tyrosine kinase substrate, a key protein in the ESCRT complex, exhibits a high degree of O-GlcNAc glycosylation that hinders the degradation of PD-L1 via the lysosomal pathway, thereby inhibiting the killing effect of T cells towards tumor cells. The combined use of OSMI-4 (another OGT inhibitor) and PD-L1 mAb can restore tumor immunity and synergistically inhibit the growth of liver cancer and melanoma in fully immunized mice, providing new ideas for PD-L1 mediated ICI therapy 9.

In summary, O-glycosylation is essential for tumor immune regulation, and its related mechanisms require further investigation. New, efficient, and safe anticancer strategies targeting O-glycosylation require continuous development.

## N-glycosylation and immunotherapy

The core of the N-linked glycan comprises two sequential GlcNAcs and three mannose molecules, which can be further extended and modified by various glycosyltransferases and glycosidases to form complex, hybrid, and high-mannose glycans 29. The increase in N-glycan branches is involved in regulating the interaction between tumor cells and the matrix and in promoting the migration of tumor cells, which is considered one of the characteristics of cancer 30.

CAR-T cell therapy targeting N-glycans has great potential for tumor immunotherapy. In this therapy, T cells from patients are genetically engineered *in vitro*, and CAR is introduced as a navigation system to activate T cells so that they can specifically recognize and attack tumor cells accurately and efficiently and improve the immune response 31. As a burgeoning anti-tumor immunotherapy, the noteworthy clinical efficacy of CAR-T cell therapy in certain malignant hematological tumors deserves acknowledgment, but the scope of clinical trials on solid tumors remains considerably limited 32. In addition to the challenges associated with antigen escape, off-target effects, and the transport and infiltration of CAR-T cells, the immunosuppressive nature of the TME may be an important factor 33. To improve the effects of cell immunotherapy and enhance the resistance of T cells to tumor cell immunosuppression, researchers have focused on the metabolic reprogramming of immune cells. The influence of N-glycans on reprogramming cannot be underestimated and requires meticulous attention. Researchers have found that N-glycans impede the effectiveness of CAR-T cells in targeting solid tumors by disrupting the formation of immune synapses, diminishing transcriptional activation, reducing cytokine production, and lowering cytotoxicity 34. 2-Deoxy-D-glucose (2DG), an analog of glucose, regulates N-glycosylation and inhibits N-glycan biosynthesis 35. After 2DG treatment, the N-glycan barrier on T cells is broken, leading to enhanced efficacy of T cell-based cancer immunotherapy 36. The anti-tumor effect of CAR-T cells was significantly enhanced when the expression of N-glycosylation was weakened by the knockout of the glycosyltransferase *MGAT5* in pancreatic cancer cells. Furthermore, combining this approach with 2DG increases the anti-tumor efficacy of CAR-T cells. This combination represents a promising strategy for overcoming the limitations of CAR-T cells in the treatment of solid tumors 34. In addition, N-glycosylation disorders are associated with immunotherapy resistance. Advanced CRC frequently exhibits primary immune escape traits, highlighting its immune resistance characteristics and explaining its poor response to IFN-y therapy or ICI 37. The glycosyltransferase MGAT3 catalyzes the formation of bisecting GlcNAc linked to mannose in the pentasaccharide core. This specific N-glycan structure is considered a metastatic suppressor that affects cell adhesion and migration. Decreased expression of MGAT3 is linked to IFN-γ resistance in CRC 38. Increasing the expression of MGAT3 can restore the sensitivity of CRC to IFN-γ or ICI treatment, representing a novel strategy to address immune-resistant CRC 39.

**Figure 1:**
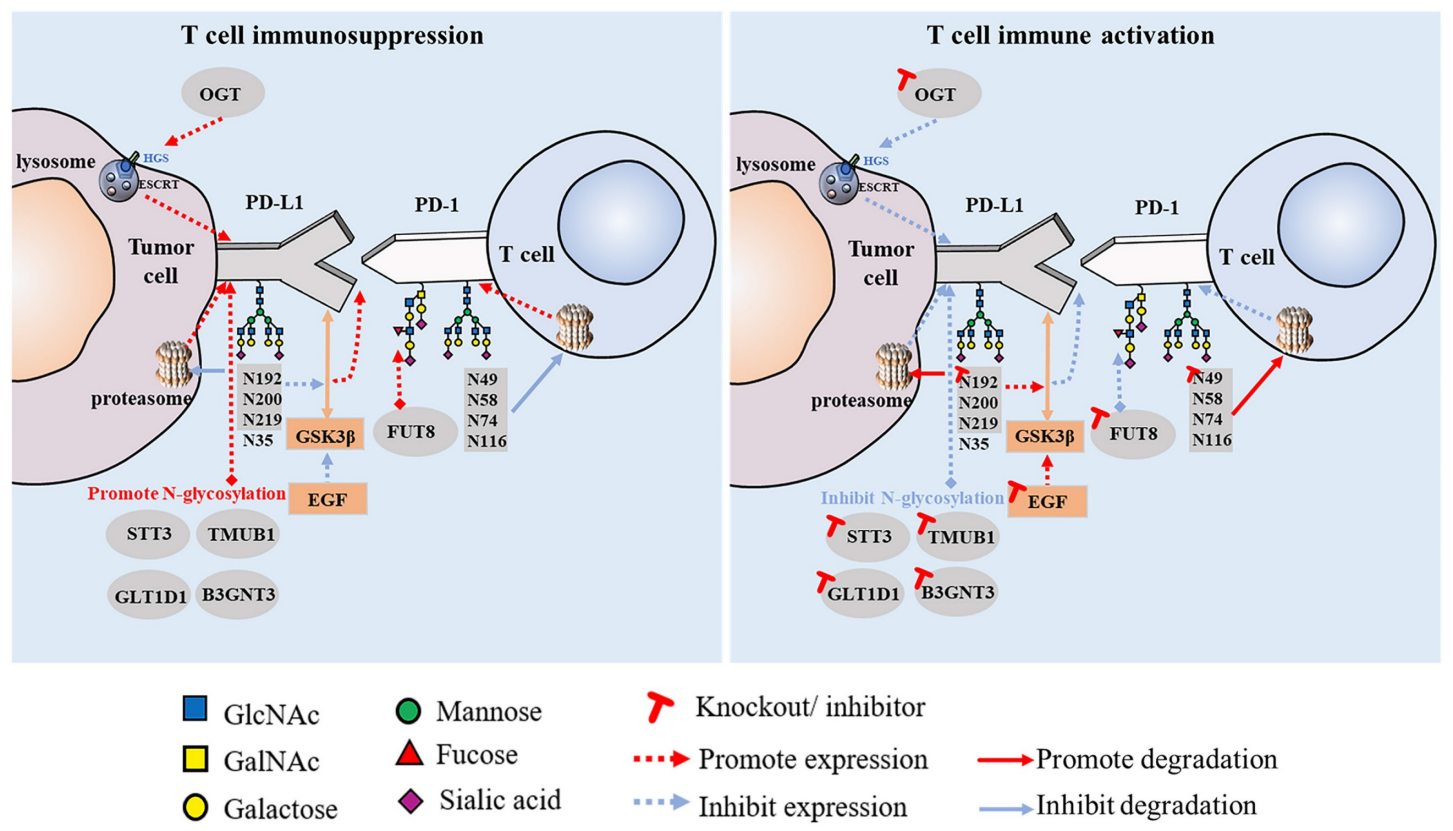
Effect of PD-1/PD-L1 glycosylation on T cell immune activity.

Increasing research has shown that glycosylation plays a vital role in the interaction between PD-1 and PD-L1. This is an important mechanism of tumor cell immune escape that significantly affects the efficacy of immunotherapy. Linking the glycosylation pathway to the strict regulation of PD-1/PD-L1 and further elucidating the molecular mechanisms related to glycosylation may provide clues for the discovery of immunotherapeutic targets for tumor therapy and new strategies to improve the efficacy of cancer immunotherapy. PD-1 is reportedly highly N-glycosylated at the N49, N58, N74, and N116 sites in T cells, which is essential for maintaining PD-1 stability and membrane expression. The glycosylation of PD-1, especially at the N58 site, plays an important role in mediating PD-L1 interaction. The mAb STM418, targeting the PD-1 N58 glycosylation site, showed higher PD-1 affinity than the previously FDA-approved nivolumab and pembrolizumab antibodies, as well as enhanced T cell anti-tumor immunity 40. The N-glycosylation of PD-L1 (N192, N200, and N219) initiates T cell immunosuppression by inhibiting 26S proteasome-mediated protein degradation and further stabilizes PD-L1 by antagonizing the interaction between PD-L1 and GSK3β 8. Glycosylated PD-L1 inhibits T cell activity in the TME, whereas non-glycosylated PD-L1 reduces immunosuppressive activity owing to its inability to bind to PD-1. Epidermal growth factor not only stabilizes PD-L1 by inhibiting GSK3β-β-TrCP-mediated degradation but also promotes the N-glycosylation of PD-L1 at N192 and N200 sites in triple-negative breast cancer (TNBC) cells by upregulating glycosyltransferase B3GNT3, thus promoting the binding of PD-L1 and PD-1 and leading to T cell failure 41. Glycosylation can also regulate the expression of PD-L1 in cancer stem cells (CSCs) 42. STT3 (catalytic subunit of the oligosaccharyltransferase complex) is upregulated by epithelial-mesenchymal transition (EMT) through the β-catenin signaling axis, thereby increasing the N-glycosylation of PD-L1, promoting the accumulation of PD-L1 in CSCs, and inducing immune escape. By targeting the EMT/β-catenin/STT3/PD-L1 axis, etoposide can further inhibit immune escape and induce the apoptosis of CSCs 43. In B-cell non-Hodgkin lymphoma (B-NHL), glycosyltransferase 1 domain-containing 1 (GLT1D1) enhances the stability of PD-L1 through N-glycosylation, thereby promoting immunosuppression and tumor growth, and is a potential target for B-NHL treatment 44. PD-L1 also has other regulatory factors, such as TGF-β1, which promotes the N-glycosylation of PD-L1 by activating the c-jun/STT3A (an isoform of STT3) signaling pathway, thereby facilitating the immune escape of nasopharyngeal carcinoma 45. Another study found that transmembrane and ubiquitin-like domain-containing protein 1 (TMUB1) is a regulator of PTM of PD-L1 in tumor cells that enhances the N-glycosylation and stability of PD-L1 by recruiting STT3A, thereby promoting PD-L1 maturation and tumor immune escape 46. When these positive PD-L1 regulators are inhibited, anti-tumor immunity can be enhanced (Figure 1).

These findings collectively suggest that the N-glycosylation of PD-L1 plays a crucial role in regulating its expression and stability, influencing tumor immune escape, and modulating the effectiveness of ICIs. Targeting molecular strategies that regulate the PTM of PD-L1 has significant clinical translational value.

## Sialylation and immunotherapy

Sialylation, the process of adding sialic acid residues at the termini of glycans, is a crucial modification that plays diverse roles in cell recognition, cell adhesion, and intercellular signal transduction 47. Tumor cells often exhibit elevated levels of sialic acid on their surfaces compared with normal cells. Owing to the significant changes in the structure and content of glycoproteins and glycolipids on the surface of cancer cells, a large amount of sialic acid may detach from the surface of cancer cells and enter the blood, resulting in an increase in serum sialic acid levels 48. Abnormal sialylation is considered a hallmark of cancer and directly affects the interaction between tumor cells and the TME, especially the interaction with sialic acid-binding immunoglobulin-type lectins (Siglecs) in the regulation of immune cell function 49. The sialoglycan-siglectin axis contributes to the formation of an immunosuppressive TME. This axis induces a tumor-promoting phenotype in TAMs, inhibits the activation of NK cells and neutrophils, reduces DC maturation and antigen presentation, and hampers T cell responses 50, 51. As a result, numerous studies have explored novel avenues for cancer treatment by strategically targeting the sialoglycan-Siglec immune axis. These studies aimed to disrupt immunosuppressive signals within the TME, offering potential therapeutic strategies to enhance anti-tumor immune responses.

Previous studies have demonstrated that the administration of sialidase to induce desialylation significantly enhances NK cell-mediated cytotoxicity and inhibits tumor progression in leukemia and cervical cancer 52. In melanoma, knockdown of the sialic acid transporter SLC35A1 can suppress the expression of sialoglycans, slow the growth rate of tumors, and promote the presence of greater quantities of effector immune cells within the TME, creating a milieu conducive to tumor immune control 53. Moreover, intratumoral injection of the selective sialoglycan biosynthesis inhibitor Ac53FaxNeu5Ac effectively interferes with the expression of sialoglycans. This intervention inhibits the growth of melanoma and 9464D neuroblastoma in mice. Notably, it also induces a shift in the composition of immune cells within the TME, favoring immune-promoting properties 54. Additional evidence supports the notion that antibody-sialidase conjugates represent a promising strategy to modulate anti-tumor immunity. In one approach, researchers coupled *Vibrio cholerae* sialidase with trastuzumab; the resulting conjugate, denoted T-Sia, demonstrated the ability to desialylate HER2+ BC cells *in vitro*, resulting in the removal of Siglec ligands and ultimately enhancing the efficacy of NK cells in killing cancer cells 55. Based on this, researchers developed a second-generation HER2 antibody-sialidase conjugate, referred to as T-Sia 2. This novel conjugate demonstrated the effective and selective removal of a variety of sialoglycans from BC cells and blocked sialoglycan-Siglec interactions. Moreover, in mice with *in vivo* transplanted homologous HER2+ BC cells, desialylation induced by T-Sia 2 was shown to decelerate tumor growth and enhance immune cell infiltration and activation within the TME 56. Recent studies have expanded our understanding of the mechanisms underlying tumor sialylation-mediated immunosuppression. High sialylation of tumor cells reportedly promotes the polarization of TAMs to an immunosuppressive phenotype by interacting with Siglec-E. Conversely, therapeutic desialylation can polarize TAMs to an immune-promoting phenotype. Notably, desialylation therapy exhibits a synergistic effect when combined with ICIs, suggesting its potential to enhance the efficacy of ICIs or alleviate drug resistance. These findings provide a robust foundation for the ongoing clinical development of sialoglycan-Siglec-targeting agents and their combination with PD-1 and CTLA-4 blocking immunotherapies 57.

Siglecs are increasingly being identified as compelling targets for tumor immunotherapy, driven by an expanding body of research that unravels the intricate mechanisms through which interactions within the sialoglycan-Siglec axis influence immune escape within the TME. Siglecs are expressed in most immune cells and play crucial roles in regulating the activity and function of cells in the innate and adaptive immune systems 58. Internal immune cells, especially macrophages, abundantly express various Siglec receptors, including Siglec-9, -10, -15, and others, which contribute to the transformation of TAMs to a cancer-promoting phenotype. Sialoglycan evades immune surveillance by binding to the inhibitory receptor Siglec-9 in the granulocyte-monocyte lineage; this is restored in a Siglec9/E-deficient mouse model, strengthening the ability of the innate immune system to effectively eliminate tumor cells 59. Another study found that Siglec-9 in primary monocytes and macrophages could induce TAM polarization to promote tumor progression by binding to abnormally sialylated MUC1 glycans in the human BC cell line T47D 60. A recent study has shown that in glioblastoma multiforme, Siglec-9 can function as an immune checkpoint molecule on the macrophage surface. This has significant implications for the efficacy of the TME and immunotherapy outcomes. Targeting Siglec-9 has been identified as a strategy to enhance the effectiveness of anti-PD-1/PD-L1 therapy 61. The highly expressed inhibitory receptor Siglec-10 on TAMs interacts with the new “don't eat me” signaling molecule CD24, activates the SHP-1/SHP-2-mediated inhibitory signaling pathway, and inhibits the phagocytosis of tumor cells by macrophages, thereby exerting an immune escape effect 62. A study published in *Nature* in 2019 showed that knockout of CD24 or Siglec-10 or the blocking of the CD24-Siglec-10 axis with mAbs significantly enhanced the phagocytosis of all human tumor cells expressing CD24 by macrophages 63. Therefore, since CD24-Siglec-10 is an innate immune checkpoint essential for mediating anti-tumor immunity, it has become a very promising therapeutic target for tumor immunotherapy. Siglec-15, which is found on the surface of both tumor cells and M2 macrophages, can effectively inhibit T cell activation to suppress anti-tumor responses. Notably, Siglec-15 has been identified as a significant immunosuppressive factor in tumors that do not express PD-L1. The mAb NC318, designed to block Siglec-15, has shown promise in restoring the anti-tumor immune effect within the TME, and a clinical trial on the efficacy of NC318 in treating solid tumors is ongoing (NCT03665285). This approach may offer a viable therapeutic option for cancer patients in whom PD-L1 immunotherapy has proven ineffective 64. Similarly, Siglec-7 and Siglec-9 are abundant in NK cells and inhibit NK cytotoxicity by interacting with sialoglycans 65. In addition, inhibitory Siglecs are involved in T cell immunoregulation. Compared to peripheral T cells from healthy donors, Siglec-9 expression was significantly upregulated in tumor-infiltrating T cells from patients with NSCLC, CRC, and OC, and targeting the sialoglycan-Siglec-9 pathway enhanced anti-tumor immunity both* in vitro* and *in vivo* 66. In addition, research on CAR-T cell therapy targeting these Siglec antigens is progressing. CAR-T cell therapy specifically targeting Siglec-2 (CD22) has demonstrated clinical effectiveness, particularly in patients with pre-B-cell acute lymphoblastic leukemia (B-ALL) who were resistant to CD19 CAR-T therapy 67. Siglec-3 (CD33)-specific CAR-T cell therapy has shown efficacy in preclinical models of acute myeloid leukemia (AML) resistance and have begun clinical trial evaluation 68. Siglec-6 is a particularly promising target because it is typically expressed in AML cell lines but not in normal hematopoietic stem and progenitor cells. The development of CAR-T cell therapy targeting Siglec-6 represents a recent breakthrough in AML treatment. Notably, this therapy is effective for treating AML without the need for subsequent allogeneic hematopoietic stem cell transplantation 69.

In recent years, the sialoglycan-Siglec axis has emerged as a potential new immune checkpoint to enhance cancer immunotherapy. The therapeutic potential of modulating this axis has been demonstrated in both related preclinical and clinical studies. Further exploration of the mechanism of sialoglycan-Siglec interaction in the TME will help to improve novel immunotherapy strategies and pave the way for further clinical applications.

## Fucosylation and immunotherapy

Fucosylation, particularly core fucosylation, is one of the most widespread cancer-related changes in the N-glycan chain 70. α-1,6-Fucosyltransferase (FUT8) is the sole known enzyme responsible for generating an α-1,6-fucosylated structure on the core residue of the N-glycan chain 71. In 2017, Okada et al. employed CRISPR-Cas9-based knock out and whole-genome sequencing technology to screen *FUT8* as a pivotal factor that regulates PD-1 expression on the cell surface. FUT8, by catalyzing PD-1 fucosylation, exerts control over PD-1 expression in T cells. The inhibition of FUT8 expression markedly diminishes core fucosylation modifications at N49 and N74 on PD-1. This reduction correlates with decreased PD-1 expression and augmented T cell activation, ultimately enhancing the efficacy of tumor eradication 72. Similarly, a study by Zhang et al. on lung adenocarcinoma confirmed that blocking the core fucosylation of PD-1 represents a viable strategy to reduce PD-1 expression in future immunotherapies 73. Furthermore, FUT8 regulates the core fucosylation of the highly glycosylated B7 homolog 3 protein (B7H3), contributing to its protein stability and immunosuppressive effects in TNBC. Knocking down *FUT8* or using 2F-Fuc (a core fucosylation inhibitor) effectively rescued B7H3-mediated immunosuppression, indicating that targeting the FUT8-B7H3 axis, particularly in combination with PD-L1, has the potential to enhance the anti-tumor immune response in patients with TNBC 74.

Previous research findings have provided support for the inhibition of the fucosylation of glycoproteins, either as a standalone treatment or in combination with ICIs, as a promising therapeutic strategy for various cancers. Ongoing phase 1 clinical trials are investigating the fucosylation inhibitor SGN-2FF in patients with advanced solid tumors. Preliminary results revealed encouraging anti-tumor activity, underscoring the potential of fucosylation inhibition as a novel avenue for cancer treatment 75.

To enhance the efficacy of various ICIs, an increasing number of studies have focused on modifying the Fc segments, primarily through glycoengineering and site-directed mutagenesis. For instance, Rony et al. employed glycoengineering to eliminate the fucose subunit (aFuc-IgG1) from the linker glycan of the Fc segment of the PD-L1 antibody. This modification led to an increased binding capacity to activated FcγRIIIA. The glycoengineered PD-L1 antibody induced a shift in neutrophils from an immunosuppressive state to a proinflammatory state in MC38 tumor-bearing mice, resulting in heightened anti-tumor activity and a more robust immune response 76. These findings suggest that defucosylation is a favorable factor for tumor immunotherapy. However, contradictions regarding the use of ACT have emerged. The efficacy of ACT in cancer treatment frequently falls short of the desired outcomes, primarily attributed to challenges associated with T cells failing to effectively home to tumor tissues 77. Alatrash *et al*. found that fucosylation, when applied *in vitro*, has the potential to augment the homing ability of cytotoxic T lymphocytes to leukemic bone marrow and tumor tissues. This enhancement amplifies the efficacy of T cells in killing tumors, ultimately improving anti-tumor outcomes 78. This underscores the complexity of fucosylation in tumor immunotherapy, challenging the simplistic classification as either favorable or unfavorable. Achieving optimal effects of targeted fucosylation requires a deeper understanding of its mechanisms of action in tumor immunology. Further exploration of these mechanisms is crucial to refine fucosylation-based strategies and maximize their therapeutic potential.

## Conclusions and perspectives

**Figure 2:**
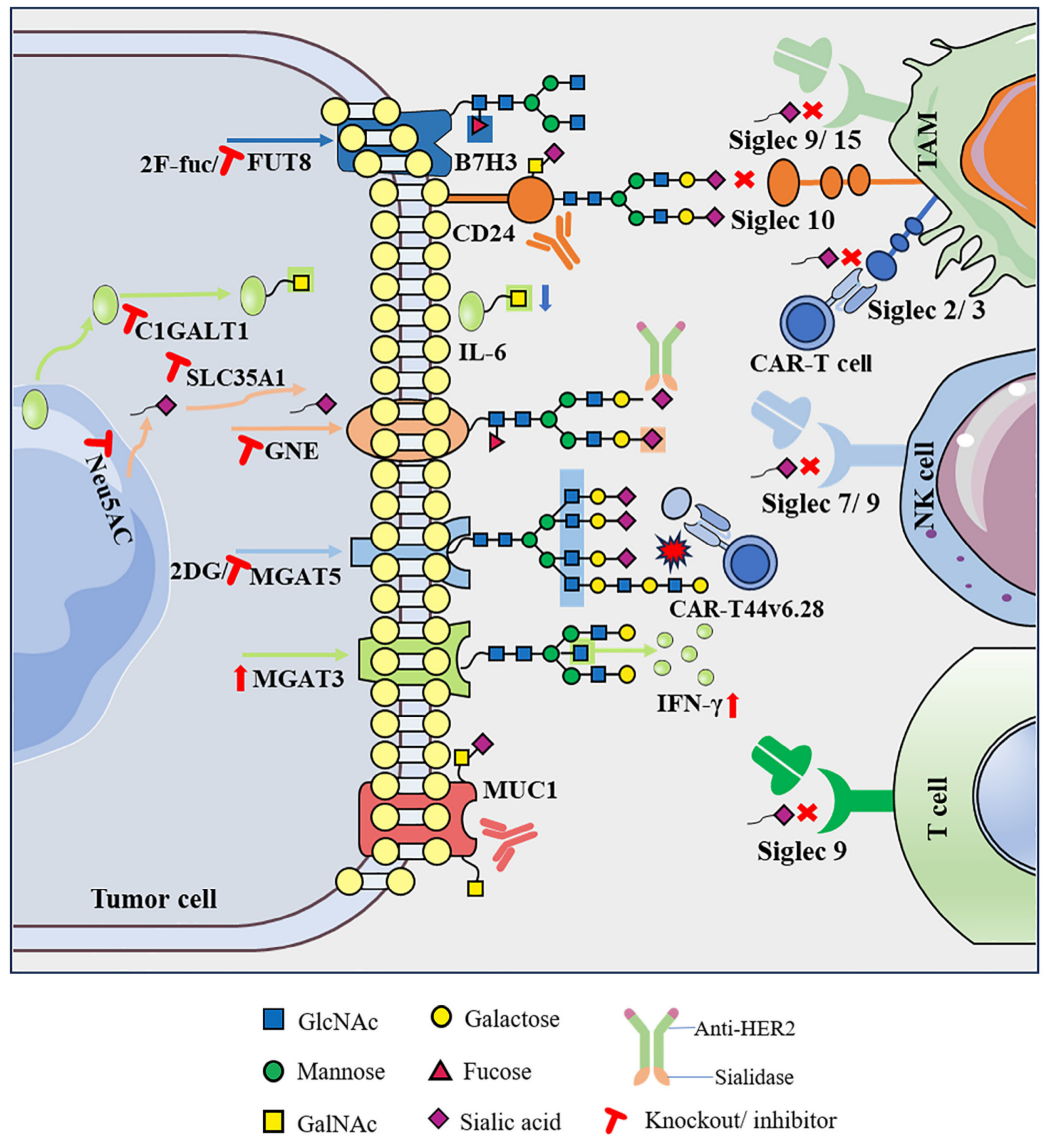
Targeting abnormal glycosylation restores the anti-tumor immune activity of immune cells.

After consolidating the vast body of literature, this review presents compelling evidence that abnormal glycosylation on the surface of tumor cells, characterized by shorter O-glycans and more branched N-glycans, along with associated alterations in the sialylation and fucosylation of glycan terminal epitopes, exerts a significant impact on cancer progression and the characteristics of the TME. Abnormal glycosylation, considered a key feature of cancer, contributes to the formation of a complex “glyco-code” on the tumor cell surface. This glyco-code not only influences self-activation but also modulates interactions with immune cells within the TME, thereby playing a crucial role in shaping the dynamics of tumor immunity. Therefore, editing glycans on the cell surface to achieve glycan reconstruction and further modifying of other biomolecules can regulate cell recognition and communication functions, representing a new breakthrough in the field of immune response restoration. First, direct targeting of abnormal glycosylation on tumor cells to eliminate the glyco-code has shown promise in reducing immunosuppressive effects. For instance, the application of sialidase or sialoglycan synthesis inhibitors interferes with sialoglycan synthesis, promoting the immune control of tumors 54-56. Second, disrupting abnormal glycosylation by modulating glycosyltransferases and glycosidases has significant therapeutic potential. For instance, knocking out *MGAT5* to eliminate defective branched N-glycans transforms CAR-T cell therapy into a potent force akin to a “cannonball” targeting and attacking pancreatic cancer cells. Additionally, inhibiting the expression of the key enzyme C1GALT1, responsible for initiating GalNAc O-glycosylation, reshapes the TME in head and neck tumors, leading to improved immunotherapy efficacy 26, 36. Third, intervention in the glycosylation-lectin pathway, specifically by directly blocking the binding of glycans to recognition molecules, as exemplified by the sialoglycan-Siglec interaction, is emerging as a potential novel immune checkpoint (Figure 2) 56.

In this new era of immunotherapy, it is crucial to emphasize enhanced response rates in patients with cancer. Most immune checkpoint molecules are glycoproteins, and their structures and biological functions are largely affected by glycosylation. Several previous studies have shown that abnormal glycosylation on the surface of immune checkpoint molecules can regulate their stability and enable tumors to evade immune surveillance. Among the extensively studied immune checkpoint molecules, PD-1 and PD-L1 undergo various types of glycosylation, including but not limited to O-GlcNAc glycosylation, which impedes lysosomal pathway-mediated PD-L1 degradation, and N-glycosylation, which inhibits PD-L1 degradation mediated by the proteasome pathway. Additionally, FUT8 catalyzes PD-1 core fucosylation, stabilizing PD-1 expression on T cells and initiating T cell immunosuppression 8, 9, 69. Some new drugs for glycosylation are undergoing *in vitro* and *in vivo* trials, whereas others have entered clinical trials. STM108 mAb targets the N192 and N200 glycosylation sites of PD-L1 and can block the interaction of PD-L1/PD-1 to promote the internalization and degradation of PD-L1. The STM108 antibody-drug conjugate, coupled with the potent anti-mitotic drug monomethyl auristatin E (MMAE), induced strong anti-tumor activity in *in vivo* and *in vitro* TNBC models 41. Similarly, STM418, MW11-h317, and MAb059c mAbs targeting the N58 glycosylation site of PD-1 can also effectively inhibit the binding of PD-1 to PD-L1 and enhance the T cell-mediated anti-tumor immune response, and these are still in the pre-clinical trial stage 40, 79, 80. SEA-TGT, an antibody targeting non-fucosylated T cell immunoreceptor with Ig and ITIM domains (TIGIT) as a new immunosuppressive molecule, was found to show significantly enhanced anti-tumor activity in previous clinical trials 81. Phase 1 clinical trials have been initiated to test the safety and efficacy of SEA-TGT in combination with anti-PD-1 antibodies in patients with advanced solid tumors and specific lymphomas 82. The glycosylation of immune checkpoint molecules has become a high-profile target for tumor immunotherapy; in particular, preclinical trials targeting the N-glycosylation of PD-1/PD-L1 have shown preliminary achievements in tumor immunotherapy. However, with in-depth study of O-glycosylation, fucosylation, sialylation, and other glycosylation processes, there is a need to develop new effective drug targets for tumor immunotherapy. In addition, abnormal glycosylation by different mechanisms leads to the formation of tumor-associated carbone antigens (TACAs), including mucin-related truncated O-glycans (Tn, sTn), gangliosides (GD2, GD3, fucosyl-GM1, GM2, and GM3), and the globular serine glycan Globo-H. Novel mAbs, antibody-drug conjugates, bispecific T cell engagers, and vaccines targeting these TACAs have entered different clinical trial stages (Tables 1 and 2), showing strong therapeutic potential in synergy with other anti-tumor strategies 83-85. Therefore, a profound exploration of the mechanism of glycosylation in the malignant process of tumors and the continuous development of anti-tumor drugs based on glycosylation are expected to provide new targets and theoretical support for personalized treatment of tumor patients.

However, owing to the complexity and diversity of glycosylation modifications, glycosylation analysis faces significant challenges. In recent years, research on glycosylation and related fields has shown the following main development trends: (1) Development and application of glycan synthesis, analysis, and editing technology: continuous innovation in mass spectrometry instrumentation coupled with the optimization of analytical and bioinformatics methods and the synthesis of standard glycoprotein/glycopeptides, which has facilitated remarkable progress. Through the integration of multi-omics datasets encompassing glycomic, genomic, and proteomic information, we can now achieve more comprehensive and accurate detection of glycosylation modifications at immune checkpoints. This holistic approach enhances our understanding of glycoprotein function and structure, thereby offering robust support for drug development and clinical treatments. (2) In-depth exploration of the molecular mechanism of abnormal glycosylation in tumors: tumor-associated abnormal glycosylation is a characteristic of cancer and plays an important role in key pathological steps such as tumor development and progression. As the regulation of abnormal glycosylation in tumor immunity has received extensive attention, an in-depth study of the molecular mechanism of glycosylation-mediated immune escape and the discovery of new effective drug targets are expected to promote the development of next-generation tumor immunotherapy. (3) Clinical transformation of tumor glycosylation drugs: At present, a considerable number of targeted tumor glycosylation drugs are in preclinical and clinical trials at different stages, showing preliminary therapeutic potential. Further efforts are needed to elucidate the mechanism of action, optimize drug molecules, and evaluate their safety and efficacy to successfully transform this strategy into a new type of tumor immunotherapy for clinical application.

In conclusion, as we have uncovered the pivotal regulatory role of abnormal glycosylation modifications in tumor cells and the intricate mechanisms linking them to immune responses, a comprehensive understanding of the significance of the glyco-code will serve as the foundation for a more refined approach to cancer immunotherapy. This presents new hope for individuals suffering from cancer, pointing towards innovative strategies that may revolutionize the cancer treatment landscape.

## Figures and Tables

**Table 1:** Clinical trials of targeted tumor glycosylation vaccines in phase 2 and subsequent research stages.

	Vaccine	Target	Type	Conditions	Phase		ClinicalTrials.gov ID	Study Start	Study Status
**1**	**Tecemotide**	MUC1	Vaccine	Lung neoplasmsNon-small cell lung carcinoma	2		NCT00157209	2000.08	Completed
				Non-small cell lung carcinomaLung neoplasms	2		NCT00157196	2005.04	Completed
				Non-small cell lung cancer	3	NCT00409188		2007.01	Completed
				Multiple myeloma	2	NCT01094548		2008.01	Completed
				Non-small cell lung cancer	1/2	NCT00960115		2008.12	Completed
				Lung cancer	2	NCT00828009		2011.01	Completed
				Colon carcinomaRectum carcinoma	2	NCT01462513		2011.08	Completed
				Prostate cancer	2	NCT01496131		2011.10	Completed
				Rectal cancer	2	NCT01507103		2012.02	Completed
**2**	**ETBX-061**	MUC1	Vaccine	Triple-negative breast cancer	1/2	NCT03387085		2018.03	Active, not recruiting
				Head and neck cancerHead and neck neoplasms	1/2	NCT04247282		2020.06	Completed
**3**	**TG4010**	MUC1	Vaccine	Non-small cell lung carcinoma	2/3	NCT00415818		2005.12	Completed
				Recurrent Stage I-IV non-small cell lung cancer	2	NCT02823990		2016.12	Completed
**4**	**CVac**	MUC1	Vaccine	Epithelial ovarian cancer	2	NCT01068509		2010.07	Completed
**5**	**ImMucin**	MUC1	Vaccine	Multiple myeloma	1/2	NCT01232712		2010.09	Completed
								
								
								
**6**	**BEC2**	GD3	Vaccine	Non-small cell lung carcinoma	3	NCT00037713		1998.09	Completed
				Lung cancer	3	NCT00006352		1999.09	Completed
**7**	**GD2L/GD3L-KLH/OPT-821**	GD2/GD3	Bivalent vaccine	Neuroblastoma	1/2	NCT00911560		2009.05	Active, not recruiting
**8**	**GM2/GD2L/GD3L-KLH/OPT-821**	GM2/GD2/GD3	Trivalent vaccine	Sarcoma	2	NCT01141491		2010.06	Completed
**9**	**Racotumomab**	NeuGcGM3	Vaccine	Advanced non-small cell lung cancer	2	NCT01240447		2009.09	Completed
				Neuroblastoma	2	NCT02998983		2016.11	Completed
**10**	**OBI-822**	Globo-H	Vaccine	Triple-negative breast cancer	3	NCT03562637		2018.12	Recruiting
**11**	**OPT-822/OPT-821**	Globo-H	Vaccine	Metastatic breast cancer	2	NCT01516307		2011.12	Completed
				Triple-negative breast cancer	3	NCT03562637		2018.12	Recruiting
**12**	**OBI-833/OBI-821**	Globo-H	Vaccine	Esophageal cancer	2	NCT05376423		2022.06	Recruiting
				Non-small cell lung cancer	2	NCT05442060		2022.07	Recruiting
**13**	**STn-KLH**	sTn	Vaccine	Breast cancer	3	NCT00003638		1999.01	Completed
				Breast neoplasms	2	NCT00046371		2002.08	Completed
									

Note: 1. Data source: https://www.clinicaltrials.gov/; 2. Clinical trials whose status was shown to be terminated and unknow were not included.

**Table 2:** Clinical trials of targeted tumor glycosylation drugs in phase 2 and subsequent research stages.

	Drug Candidate	Target	Drug Type	Conditions	Phase	ClinicalTrials.gov ID	Study Start	Study Status
**1**	**Moxetumomab pasudotox**	CD22	ADC	Hairy cell leukemia	2	NCT00923013	2008.10	Active, not recruiting
				Non-Hodgkin lymphomaChronic lymphocytic leukemia	2	NCT01030536	2010.02	Completed
				Hairy cell leukemia	3	NCT01829711	2013.04	Completed
**2**	**Inotuzumab ozogamicin**	CD22	ADC	B-cell lymphoma	1/2	NCT00299494	2006.05	Completed
				B-cell lymphoma	2	NCT00867087	2009.06	Completed
				Lymphoma	2	NCT00868608	2009.07	Completed
				Acute lymphoblastic leukemia	2	NCT01134575	2010.06	Completed
				B acute lymphoblastic leukemia with t(9;22)(q34.1;q11.2); BCR-ABL1;B acute lymphoblastic leukemia, Philadelphia chromosome negativeBurkitt-like lymphoma with 11q aberrationHigh grade B-cell lymphoma with MYC and BCL2 and/or BCL6 rearrangementsHigh grade B-cell lymphoma, not otherwise specifiedRecurrent B acute lymphoblastic leukemiaRecurrent Burkitt lymphomaRefractory B acute lymphoblastic leukemiaRefractory Burkitt lymphoma	1/2	NCT01371630	2011.08	Recruiting
				Acute lymphocytic leukemia	2	NCT01363297	2011.08	Completed
				Acute lymphoblastic leukemia	3	NCT01564784	2012.08	Completed
				Hematopoietic and lymphoid cell neoplasm	1/2	NCT01664910	2012.10	Completed
				Diffuse large B-cell lymphoma	1/2	NCT01562990	2012.12	Completed
				Diffuse large B-cell lymphoma	2	NCT01679119	2013.10	Completed
				B acute lymphoblastic leukemia with t(9;22)(q34.1;q11.2); BCR-ABL1Blast phase chronic myelogenous leukemia, BCR-ABL1 PositiveBlasts more than 5 percent of bone marrow nucleated cellsPhiladelphia chromosome-positive, BCR-ABL1-positive chronic myelogenous leukemiaRecurrent chronic myelogenous leukemia, BCR-ABL1 positiveRefractory chronic myelogenous leukemia, BCR-ABL1 positive	1/2	NCT02311998	2015.04	Completed
				B acute lymphoblastic leukemiaB lymphoblastic leukemia	2	NCT02877303	2016.11	Recruiting
				Recurrent B acute lymphoblastic leukemia Recurrent B lymphoblastic leukemiaRefractory B acute lymphoblastic leukemiaRefractory B lymphoblastic lymphoma	2	NCT02981628	2017.06	Active, not recruiting
				Acute lymphocytic leukemia	1/2	NCT03104491	2017.07	Recruiting
				Acute lymphoblastic leukemia-Ph-negative CD22+ B-cell precursor	2	NCT03249870	2017.12	Active, not recruiting
				Acute lymphoblastic leukemiaB acute lymphoblastic leukemiaRecurrent B acute lymphoblastic leukemia	2	NCT03441061	2018.02	Recruiting
				Precursor cell lymphoblastic leukemia	2	NCT03460522	2018.05	Recruiting
				Acute lymphoblastic leukemia Hyperbilirubinemia	2	NCT03564678	2018.05	Recruiting
				Acute lymphoblastic leukemia	2	NCT03913559	2019.05	Recruiting
				B acute lymphoblastic leukemia, Philadelphia chromosome negativeRecurrent B acute lymphoblastic leukemiaRefractory B acute lymphoblastic leukemia	2	NCT03739814	2019.05	Recruiting
				Leukemia, precursor B-cell lymphoblasticLeukemia-lymphoma;Acute lymphoblastic leukemia	4	NCT03677596	2019.07	Completed
				Acute lymphoblastic leukemiaB acute lymphoblastic leukemiaLymphocytic neoplasmLymphoma	2	NCT03856216	2019.10	Recruiting
				B acute lymphoblastic leukemiaB lymphoblastic leukemiaCentral nervous system leukemiaMixed phenotype acute leukemiaTesticular leukemia	3	NCT03959085	2019.10	Recruiting
				Leukemia, acute lymphoblastic	3	NCT04307576	2020.07	Recruiting
				B acute lymphoblastic leukemia with t(9;22)(q34.1;q11.2); BCR-ABL1	3	NCT04530565	2021.01	Recruiting
				Lymphoblastic leukemiaAcute lymphoblastic leukemiaPh+ acute lymphoblastic leukemia	2	NCT04747912	2021.03	Recruiting
				Acute lymphocytic leukemia	2	NCT05456698	2022.08	Not yet recruiting
				ALLMRD-positive	2	NCT05940961	2022.08	Recruiting
				Acute lymphoblastic leukemia	4	NCT05687032	2023.02	Not yet recruiting
				B acute lymphoblastic leukemia B lymphoblastic lymphoma	2	NCT05303792	2023.02	Recruiting
				Acute lymphoblastic leukemia	2	NCT05748171	2023.05	Recruiting
				Leukemia	2	NCT05645718	2023.07	Recruiting
				Acute lymphoblastic leukemia	1/2	NCT06087419	2023.10	Not yet recruiting
				Relapsed/refractory B-cell acute lymphocytic leukemia	1/2	NCT06287229	2024.08	Not yet recruiting
**3**	**Epratuzumab**	CD22	ADC	LeukemiaLymphoma	1/2	NCT00004107	1998.02	Completed
				LeukemiaLymphoma	1/2	NCT00004084	1998.04	Completed
				Non-Hodgkin's lymphomaLymphoma, B-Cell	1/2	NCT00061425	2000.08	Completed
				NHLB-cell NHLNon-Hodgkin's Lymphoma	1/2	NCT00421395	2002.08	Completed
				Recurrent childhood acute lymphoblastic leukemia	2	NCT00098839	2005.02	Completed
				Lymphoma	2	NCT00301821	2006.01	Completed
				Non-Hodgkin's lymphoma	2	NCT00044902	2007.12	Completed
				Lymphoma	2	NCT00553501	2008.03	Completed
				Leukemia	2	NCT00945815	2010.09	Completed
				B ALL;CD22+ expressionRefractory B-ALL	2	NCT01219816	2010.11	Completed
				Acute lymphoblastic leukemia (ALL)	3	NCT01802814	2014.05	Completed
				Blasts 5 percent or more of bone marrow nucleated cellsCD22 positivePhiladelphia chromosome positiveRecurrent B acute lymphoblastic leukemiaRefractory B acute lymphoblastic leukemia	1/2	NCT03698552	2018.08	Recruiting
**4**	**Gemtuzumab ozogamycin**	CD33	ADC	Leukemia, myelocytic, acute	3	NCT00962767	2002.05	Completed
				Leukemia, myelocytic, acute	3	NCT00136084	2002.08	Completed
				Leukemia	3	NCT00052299	2002.09	Completed
				Leukemia	3	NCT00049517	2002.12	Completed
				Acute myeloid leukemia	3	NCT00476541	2004.01	Completed
				Leukemia	3	NCT00085709	2004.07	Completed
				Leukemia;Myelodysplastic syndromes	3	NCT00121303	2005.01	Completed
				Leukemia	3	NCT00372593	2006.08	Completed
				Leukemia; myelodysplastic syndromes	2/3	NCT00454480	2006.08	Completed
				Leukemia	3	NCT00492856	2007.06	Completed
				Acute myeloid leukemia	3	NCT00860639	2007.10	Completed
				Acute myeloid leukemia	3	NCT00927498	2007.12	Completed
				Acute myeloid leukemia	2/3	NCT00909168	2008.03	Completed
				Acute myeloid leukemia	3	NCT00893399	2010.05	Completed
				Acute myeloid leukemia	2/3	NCT02473146	2015.11	Completed
**5**	**Lintuzumab**	CD33	ADC	Leukemia	2	NCT00002609	1994.08	Completed
				LeukemiaMyelodysplastic syndromesNeutropenia	2	NCT00002800	1996.07	Completed
				Leukemia	2	NCT00016159	2000.01	Completed
				Acute myeloid leukemia	2	NCT00528333	2007.09	Completed
**6**	**Abagovomab**	MUC1	mAbs	Ovarian cancerFallopian tube neoplasmsPeritoneal neoplasms	1/2	NCT00103545	2003.07	Completed
**7**	**BM7PE**	MUC1	ADC	Colorectal cancer metastatic	1/2	NCT04550897	2020.08	Recruiting
**8**	**Oregovomab**	MUC16	mAbs	Ovarian neoplasms	2	NCT01616303	2012.06	Completed
				Pancreatic adenocarcinomaResectable pancreatic carcinomaStage I-III pancreatic cancer	2	NCT01959672	2013.09	Completed
				Carcinoma, ovarian epithelialOvarian neoplasmsOvarian cancerOvarian serous adenocarcinomaFallopian tube neoplasmsFallopian tube adenocarcinomaFallopian tube serous adenocarcinomaPeritoneal cancerPeritoneal carcinomaPeritoneal neoplasms	3	NCT04498117	2020.08	Active, not recruiting
**9**	**REGN-4018**	MUC16/CD3	BiTE	Recurrent ovarian cancerRecurrent fallopian tube cancerRecurrent primary peritoneal cancerRecurrent endometrial cancer	1/2	NCT03564340	2018.05	Recruiting
				Ovarian cancerFallopian tube cancerPrimary peritoneal cancer	1/2	NCT04590326	2020.12	Recruiting
**10**	**REGN-5668**	MUC16/CD28	BiTE	Ovarian cancerFallopian tube cancerPrimary peritoneal cancer	1/2	NCT04590326	2020.12	Recruiting
**11**	**Dinutuximab**	GD2	mAbs	High-risk neuroblastoma	3	NCT01041638	2009.12	Completed
				Neuroblastoma, recurrent	2	NCT02258815	2010.08	Completed
				Neuroblastoma	1/2	NCT01592045	2012.08	Completed
				GanglioneuroblastomaRecurrent neuroblastoma	2	NCT01767194	2013.02	Completed
				Metastatic malignant neoplasm in the lungMetastatic osteosarcomaRecurrent osteosarcoma	2	NCT02484443	2016.02	Completed
**12**	**Naxitamab**	GD2	mAbs	Recurrent osteosarcoma	2	NCT02502786	2015.07	Recruiting
				Neuroblastoma	2	NCT03363373	2018.04	Recruiting
				Neuroblastoma recurrent	2	NCT05754684	2022.01	Recruiting
				High-risk neuroblastoma	2	NCT05489887	2022.09	Recruiting
				Neuroblastoma	4	NCT05421897	2022.10	Recruiting
				Neuroblastoma	2	NCT06013618	2023.06	Recruiting
				Ewing sarcoma	2	NCT05968768	2023.10	Recruiting
				Neuroblastoma	4	NCT06047535	2023.10	Active, not recruiting
				Anatomic stage IV breast cancer AJCC v8HER2-negative breast carcinoma	1/2	NCT06026657	2024.03	Recruiting
**13**	**Ecromeximab**	GD3	mAbs	Metastatic melanomaCutaneous melanoma	2	NCT00679289	2008.03	Completed
**14**	**BMS-986012**	FucGM1	mAbs	Small cell lung cancer	1/2	NCT02247349	2014.11	Completed
				Extensive-stage small cell lung cancer	2	NCT04702880	2021.03	Recruiting
**15**	**PankoMab-GEX™**	Tn	mAbs	Ovarian epithelial cancer recurrentFallopian tube cancerPrimary peritoneal cancer	2	NCT01899599	2013.09	Completed

Note: 1. Data source: https://www.clinicaltrials.gov/;2. Clinical trials whose statuses were shown to be terminated and unknown were not included;3. The clinical trials of gemtuzumab ozogamycin are numerous, with only phase 3 and subsequent clinical trials listed in the table.

## Abbreviations

TME: tumor microenvironment
mAbs: monoclonal antibodies
ICIs: immune checkpoint inhibitors
ACT: adoptive cell transfer therapy
CAR-T: chimeric antigen receptor T
PTM: post-translational modification
TAMs: tumor-associated macrophages
NK cells: natural killer cells
DC: dendritic cell
Siglecs: sialic acid-binding immunoglobulin-type lectins
OC: ovarian cancer
NSCLC: non-small cell lung cancer
GalNAc: N-acetylgalactosamine
TACAs: tumor-associated carbohydrate antigens
BC: breast cancer
TNBC: triple negative breast cancer
AML: acute myeloid leukemia
CRC: colorectal cancer
GlcNAc: N-acetyl-D-glucosamine
ECSCs: esophageal carcinoma stem cells
ESCRT: endosomal sorting complexes required for transport
2DG: 2-Deoxy-D-glucose
CSCs: cancer stem cells
EMT: epithelial-mesenchymal transition
B-NHL: B-cell Non-Hodgkin's lymphoma
MMAE: monomethyl auristatin E
TIGIT: T cell immunoreceptor with Ig and ITIM domains
ADC: antibody-drug conjugates
BiTE: Bispecific T cell engager
